# Pregnancy Increases CYP3A Enzymes Activity as Measured by the 4β-Hydroxycholesterol/Cholesterol Ratio

**DOI:** 10.3390/ijms232315168

**Published:** 2022-12-02

**Authors:** Eulambius M. Mlugu, Omary M. Minzi, Appolinary A. R. Kamuhabwa, Ulf Diczfalusy, Eleni Aklillu

**Affiliations:** 1 Division of Clinical Pharmacology, Department of Laboratory Medicine, Karolinska Institutet at Karolinska University Hospital, Huddinge, 14186 Stockholm, Sweden; 2 Department of Pharmaceutics and Pharmacy Practice, School of Pharmacy, Muhimbili University of Health and Allied Sciences, Dar es Salaam P.O. Box 65013, Tanzania; 3 Department of Clinical Pharmacy and Pharmacology, School of Pharmacy, Muhimbili University of Health and Allied Sciences, Dar es Salaam P.O. Box 65013, Tanzania; 4 Division of Clinical Chemistry, Department of Laboratory Medicine, Karolinska Institutet at Karolinska University Hospital, 14186 Stockholm, Sweden

**Keywords:** pregnancy, trimester, CYP3A, 4β-hydroxycholesterol/cholesterol

## Abstract

Changes in cortisol and other hormones during pregnancy may alter CYP3A enzymes activity, but data from sub-Saharan Africa are sparse. We investigated the effect of pregnancy and *CYP3A5* genotypes on CYP3A enzymes activity using the plasma 4β-hydroxycholesterol (4β-OHC)/cholesterol (Chol) ratio, a known endogenous biomarker. Tanzanian pregnant women (*n* = 110) and non-pregnant women (*n* = 59) controls were enrolled. Plasma 4β-OHC and Chol were determined in the second and third trimesters for pregnant women and once for non-pregnant women using gas chromatography–mass spectrometry. Genotyping for *CYP3A5* (*3, *6, *7) was performed. Wilcoxon Signed-Rank Test and Mann–Whitney U test were used to compare the median 4β-OHC/Chol ratio between trimesters in pregnant women and between pregnant and non-pregnant women. Repeated-measure ANOVA was used to evaluate the effect of the *CYP3A5* genotypes on the 4β-OHC/Chol ratio in pregnant women. No significant effect of the pregnancy status or the *CYP3A5* genotype on the cholesterol level was observed. The plasma 4β-OHC/Chol ratio significantly increased by 7.3% from the second trimester to the third trimester (*p* = 0.02). Pregnant women had a significantly higher mean 4β-OHC/Chol ratio than non-pregnant women, (*p* < 0.001). In non-pregnant women, the mean 4β-OHC/Chol ratio was significantly lower in carriers of defective *CYP3A5* alleles (*3, *6 or *7) as compared to women with the CYP3A5*1/*1 genotypes (*p* = 0.002). Pregnancy increases CYP3A enzymes activity in a gestational-stage manner. The *CYP3A5* genotype predicts CYP3A enzymes activity in the black Tanzanian population, but not during pregnancy-mediated CYP3A enzyme induction.

## 1. Introduction

More than 50% of various clinically used medicines are metabolized by cytochrome P4503A (CYP3A) enzymes [1]. Monitoring changes in CYP3A enzymes activity is important, especially in efficacy studies and in studies evaluating drug–drug interaction. Midazolam clearance [2], urine 6β-hydroxycortisol/cortisol ratio [3] and dextromethorphan/3-hydroxymorphinan ratio [4] are common in vivo biomarkers for determining CYP3A enzymes activity. Midazolam is considered a gold standard as a probe drug among CYP3A4/3A5 substrates for evaluating CYP3A activity [5].

The use of probe drugs may have limited application in some populations including pregnant women. The compound 4β-hydroxycholesterol (4β-OHC), a cholesterol (Chol) metabolite formed by the CYP3A4/CYPA5 metabolic pathway, has emerged as an efficient endogenous biomarker for determining the CYP3A enzyme phenotype [6]. The ratio 4β-OHC/Chol rather than the plasma 4β-OHC level alone is considered a precise indicator of CYP3A enzyme activity. The use of this endogenous biomarker (4β-OHC/Chol ratio) with minimal blood sampling is thus suitable even for pregnant women, for whom probe drugs may be contraindicated.

The nervous system governs the physiological regulation of CYP enzymes via hormonal pathways involving growth hormone, sex hormones, cortisol/corticosterone and thyroid hormones [7]. The hormones act directly or indirectly on the hepatic nuclear/cytosolic receptors regulating the transcription of CYP genes and consequently the expression of the respective CYP enzymes. During pregnancy, the levels of cortisol, estrogens, progesterone and growth hormones increase substantially [8], leading to a potential increase in CYP3A enzymes activity. In addition, physiological changes during pregnancy including reduced intestinal motility, increased plasma volume, altered gastric pH and protein binding of drugs may also contribute to altered drug disposition and pharmacokinetics. Various chronic conditions require the continuous use of medication during pregnancy. For instance, antiretroviral drugs are used for the prevention of the mother-to-child transmission (PMTCT) of HIV during pregnancy. Significant effects of pregnancy on CYP3A activity may put pregnant women and newborns at risk. A significant increase in CYP3A enzymes by rifampicin and efavirenz was reported [9].

A modeling study indicates that CYP3A enzymes activity increases from the first to the third trimester [10]. The impact of pregnancy on the activity of the CYP3A enzymes may result in altered clinical outcomes [11], indicating the need for a continuous monitoring of drug–drug interaction and of the clinical efficacy of drugs during pregnancy. Given the advantages over midazolam, the 4β-OHC/Chol ratio can be used in clinical studies involving pregnant women. Nevertheless, data on the use of the 4β-OHC/Chol ratio as an endogenous biomarker for CYP3A phenotype in pregnant women are scarce. Two studies, albeit with small sample sizes, reported significant increases in CYP3A enzymes activity in Caucasian pregnant women compared to postpartum and non-pregnant women determined by the 4β-OHC/Chol ratio [12,13]. The only study which evaluated the effect of the trimester reported a non-significant difference in CYP3A enzymes activity between trimesters [12]. The limited literature indicates a dire need for more studies with a relatively larger sample size to provide further evidence on the effect of pregnancy and trimesters on the CYP3A phenotype using the 4β-OHC/Chol ratio.

*CYP3A4* is genetically polymorphic, with several variant alleles characterized so far. Notwithstanding, the contemporary knowledge implies no significant contribution of *CYP3A4* genetic differences on the reported variability in the constitutive CYP3A activity [14]. The reason could be either that the variants are relatively not well established or that they have limited functional significance. On the other hand, the *CYP3A5* genotype may be the most important genetic contributor to inter-individual differences in CYP3A-dependent drug clearance, possibly due to its polymorphic nature and the existence of various defective variants alleles exhibiting wide inter-ethnic variations.

A functional CYP3A5 enzyme is expressed in about 70% of the black African population [15], in contrast to European populations, where 80–90% of the individuals are homozygous for the non-functional CYP3A5*3 allele [16]. Studies involving non-pregnant populations reported contrasting effects of *CYP3A5* genetic variations on the plasma 4β-OHC/Chol ratio. Some reported a significant impact [17,18], whereas other reported no significant impact [19]. To the best of our knowledge, there are no data on the effect of *CYP3A5* genetic variations on the 4β-OHC/Chol ratio among pregnant women. The high proportion of individuals expressing *CYP3A5* in the black African populations further suggests the need to investigate the role of *CYP3A5* genetic variations on CYP3A enzymes activity using the 4β-OHC/Chol ratio among pregnant women.

The present study reports the effects of pregnancy, trimesters and *CYP3A5* genotypes on CYP3A enzymes activity as determined by the plasma 4β-OHC/Chol ratio.

## 2. Results

### 2.1. Baseline Characteristics of the Participants

A total of 110 pregnant women and 59 non-pregnant women controls from Tanzania were recruited for this study. There was no significant difference in median age and body weight between the two groups. About half of the pregnant women had been pregnant three times or more (multigravida). Table 1 presents the participants baseline characteristics.

### 2.2. Change in the 4β-OHC/Chol Ratio during Pregnancy

**Figure 1:**
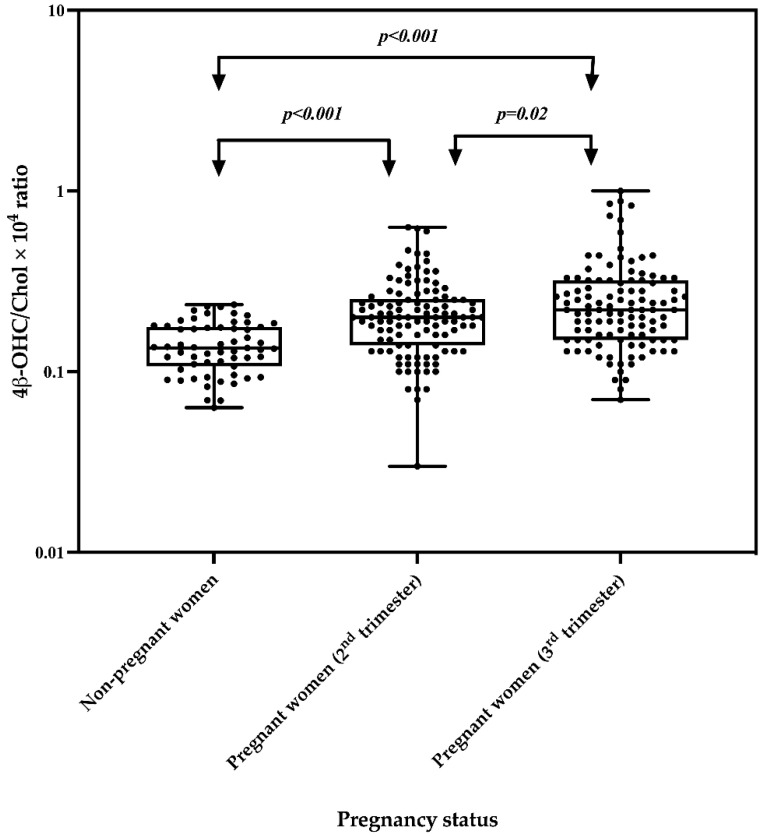
Comparison of the geometric mean of the 4β-OHC/Chol × 10^4^ ratio between non-pregnant and pregnant women in the second and third trimesters. The box plots show the mean ± SD, while whiskers denote the minimum and maximum values.

The plasma 4β-OHC and Chol concentrations were measured in the second and third trimesters among pregnant women. Table 2 shows the median with interquartile range (IQR) for 4β-OHC, Chol and the 4β-OHC/Chol ratio, with their respective median percent change from the second to the third trimesters. Compared to the second trimester the median 4β-OHC and 4β-OHC/Chol ratio were significantly higher in the third trimester, with a median percent change of 9% and 7.3%, respectively (Table 2). The 4β-OHC/Chol ratio increased significantly from the second trimester to the third trimester, *p* = 0.02 (Figure 1). There was no significant difference in the median Chol levels between the second and the third trimesters (Table 2).

### 2.3. Effect of Pregnancy on the 4β-OHC/Chol Ratio

Plasma concentrations of 4β-OHC and Chol and the 4β-OHC/Chol ratio in pregnant women were compared with their levels in non-pregnant women. The median 4β-OHC and 4β-OHC/Chol ratio were significantly higher in pregnant women, both in the second and in the third trimester, as compared to non-pregnant women (Table 3). The geometric mean of the 4β-OHC/Chol ratio was also significantly higher in pregnant women both in the second and in the third trimester, as compared to non-pregnant women, indicating a higher CYP3A activity in the former (Figure 1). In addition, the log_10_ 4β-OHC/Chol was significantly lower by 0.16 (Beta coefficient (95% CI): −0.16 (−0.22 to −0.10) *p* < 0.001) and by 0.23 (Beta coefficient (95% CI): −0.23 (−0.29–−0.16) *p* < 0.001) in non-pregnant women as compared to pregnant women in the second and third trimesters, respectively.

### 2.4. Effect of the CYP3A5 Genotype on the 4β-OHC/Chol Ratio

The allele frequencies for CYP3A5*3, CYP3A5*6 and CYP3A5*7 were 19%, 20% and 13%, respectively, with no significant differences between observed and expected genotype frequencies according to the Hardy–Weinberg equilibrium.

**Figure 2:**
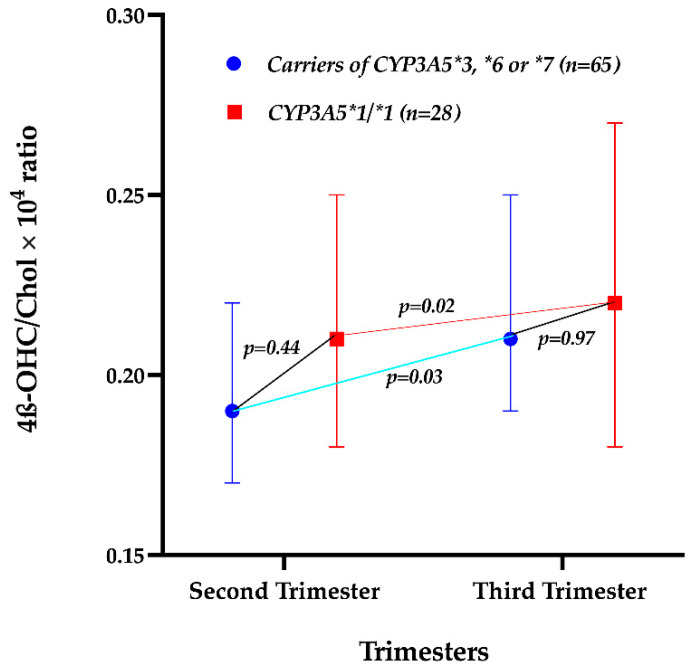
Comparison of the geometric mean of the 4β-OHC/Chol × 10^4^ ratio between *CYP3A5* genotypes among pregnant women in the second and third trimesters. The red squares indicate the geometric mean of the 4β-OHC/Chol × 10^4^ ratio in women with CYP3A5*1/*1 genotypes, while the blue dots indicate the geometric mean of the 4β-OHC/Chol × 10^4^ ratio in carriers of defective CYP3A5*3*6*7 alleles in the second and third trimesters. The whiskers indicate the 95% confidence interval of the means.

The impact of the *CYP3A5* genotype on the 4β-OHC/Chol ratio among pregnant and non-pregnant women was evaluated. The *CYP3A5* genotype did not significantly predict the change in Log_10_ 4β-OHC/Chol during pregnancy (*p* = 0.60). Further analysis revealed no significant difference in the geometric mean of the 4β-OHC/Chol ratio between pregnant women carrying defective *CYP3A5* variant alleles and those with two functional *CYP3A5* alleles (CYP3A5*1/*1) (Figure 2). The geometric mean of the 4β-OHC/Chol ratio increased significantly from the second to the third trimester both in pregnant women with zero or one functional *CYP3A5* allele and in those with two functional *CYP3A5* alleles (Figure 2).

**Figure 3:**
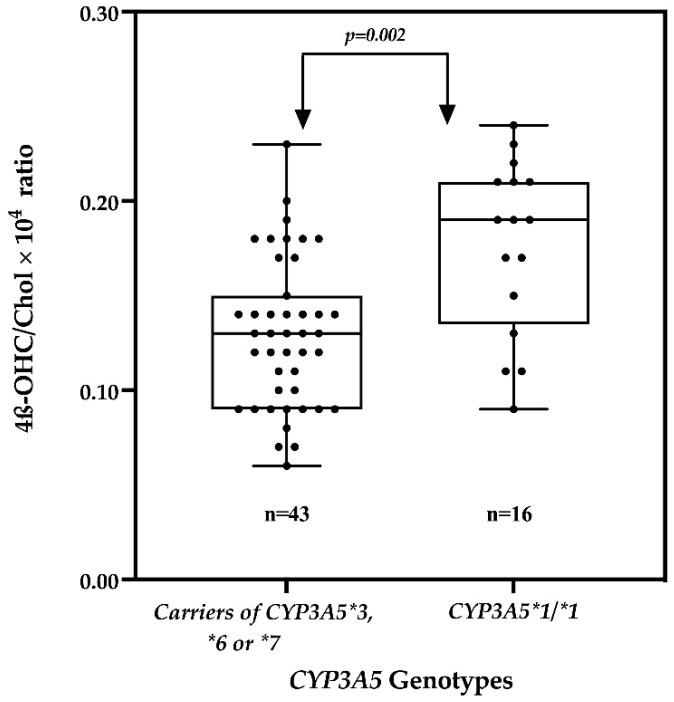
Comparison of the geometric mean of the 4β-OHC/Chol × 10^4^ ratio between *CYP3A5* genotypes among non-pregnant women. The box plot shows the mean ± SD, while the whiskers denote the minimum and maximum values.

On the other hand, the *CYP3A5* genotype predicted the plasma 4β-OHC/Chol ratio significantly in non-pregnant women. The geometric mean of the 4β-OHC/Chol ratio among non-pregnant women was significantly lower in those with zero or one functional *CYP3A5* allele as compared to those with two functional alleles (Figure 3). Furthermore, non-pregnant women with zero or one functional *CYP3A5* allele had significantly lower log_10_4β-OHC/Chol by 0.13 (adjusted Beta coefficient = −0.13 (95% CI: −0.21 to −0.05, *p* = 0.002) as compared to women with two functional allele.

## 3. Discussion

Various studies using probes suggested that CYP3A enzymes activity is increased during pregnancy [11,20]. However, drug markers for measuring CYP3A enzymes activity have some limitations in pregnant women [21]. Evidence indicates that the 4β-OHC/Chol ratio, an endogenous biomarker, can best predict the CYP3A enzyme phenotypes in clinical studies [22]. Nevertheless, data regarding the use of the 4β-OHC/Chol ratio for measuring CYP3A enzymes activity in pregnant women are limited in the literature. The present study evaluated the effect of pregnancy, trimesters and *CYP3A5* genetic variations on CYP3A enzyme phenotypes using the 4β-OHC/Chol ratio. To the best of our knowledge, this is the second study to investigate the effect of the trimester on CYP3A enzymes activity but the first to report significantly effects of the trimester on CYP3A enzymes activity in a relatively large cohort using the 4β-OHC/Chol ratio. The main findings of this study include (i) a significantly higher 4β-OHC/Chol ratio indicating increased CYP3A enzymes activity in pregnant women compared to non-pregnant women, (ii) a significantly higher 4β-OHC/Chol ratio indicating increased CYP3A enzymes activity in the third trimester compared to the second trimester and (iii) a significantly higher 4β-OHC/Chol ratio indicating higher enzymes activity in non-pregnant women with two functional *CYP3A5* alleles as compared to those with zero or one functional *CYP3A5* allele.

The findings of this study indicate that CYP3A enzymes activity was increased in pregnant women as indicated by the significantly higher 4β-OHC/Chol ratio both in the second and in the third trimester as compared to non-pregnant women. Both Chol and 4β-OHC are highly bound to plasma proteins [23]. During pregnancy, plasma proteins decrease [24], and consequently the binding of Chol and 4β-OHC may be decreased, leading to increased concentrations of free Chol and 4β-OHC in the plasma. However, we did not find significant differences in plasma cholesterol levels between pregnant and non-pregnant women, suggesting that the significant increase in the 4β-OHC/Chol ratio was solely due to an induction effect of pregnancy on CYP3A enzymes. Hormones, such as cortisol, sex and growth hormone are involved in the physiological regulation of CYP3A expression [7]. The increasing levels of these hormones during pregnancy and other physiological changes [8] might explain the observed increased CYP3A enzymes activity. Using the 4β-OHC/Chol ratio as a biomarker, a study from Sweden reported a significant increase in CYP3A enzymes activity among pregnant women at delivery as compared to women four weeks postpartum [13]. Similarly, a study from South Korea reported a significant increase in CYP3A enzymes activity in pregnant women in all trimesters as compared to non-pregnant women [12]. The findings of the present study in black African women are comparable to those of the two studies conducted in Sweden [13] and South Korea [12], despite the previously reported interethnic differences in CYP3A enzymes activity between the three countries [25]. Taken together, these findings corroborate physiological pharmacokinetics models [10,26] and clinical studies which used standard probe drugs [4,27]. Thus, the present study adds evidence indicating that CYP3A enzymes are induced during pregnancy and that the 4β-OHC/Chol ratio is a useful biomarker for monitoring CYP3A activity in pregnant women.

Though CYP3A is also expressed in the intestinal mucosa, the 4β-OHC/Chol ratio reflects only the hepatic CYP3A activity similar to other endogenous CYP3A biomarkers [28]. The regulation of CYP3A enzymes expression in the liver and in the small intestine is similar [29], and likely, the pregnancy-related increase in hepatic CYP3A enzyme activity may also be reflected in the intestine. Nevertheless, the role of intestinal CYP3A in the systemic metabolism of cholesterol is currently unknown. Compared to the hepatic cholesterol metabolism, the function of intestinal CYP3A in the formation of 4β-OHC may probably be negligible, considering that only a fraction of total mesenteric arterial blood flow permeates the mucosal villi.

This study reports increased CYP3A enzymes activity in the third compared to the second trimester, as indicated by the significantly higher 4β-OHC/Chol ratio in the third compared to the second trimester. The study from South Korea evaluating the effect of the trimester on CYP3A enzymes activity using the 4β-OHC/Chol ratio reported a non-significant increase in CYP3A activity in all trimesters [12], contrary to the present study. One reason to explain the observed difference might be the relatively large sample size in the present study which was three times larger than that of the study conducted in South Korea [12]. In addition, the difference in median gestation age in the second trimester, which was lower by three weeks in our study compared to the study performed in south Korea, might also explain the observed difference. Nevertheless, the findings of our study corroborate findings of physiological pharmacokinetic models. We recommend more powered studies to validate the impact of the trimester on CYP3A enzymes activity using the 4β-OHC/Chol ratio.

We observed that non-pregnant women with zero or one functional *CYP3A5* alleles had a lower enzymes activity, as indicated by a significantly lower geometric mean of the 4β-OHC/Chol as ratio compared to women with two functional alleles. Our finding agrees with the fact that single-nucleotide polymorphisms in *CYP3A5* decreases the enzymes’ phenotypic activity. The findings of this study are comparable to those of other previous studies conducted in non-pregnant populations. A study among twins in Germany reported significantly lower 4β-OHC levels among individuals with homozygous CYP3A5*3 as compared to those with functional CYP3A5*1 [17]. Similarly, a study conducted in Ethiopia reported a significantly lower geometric mean of the 4β-OHC/Chol ratio among individuals with zero or one functional *CYP3A5* alleles as compared to those with two functional *CYP3A5* alleles [18]. Other studies reported non-significant effects of *CYP3A5* genetic variations on the 4β-OHC/Chol ratio [19], contrary to our study. Nevertheless, the findings of this study suggest the clinical importance of the *CYP3A5* genotype in black populations, where the enzyme is expressed to a larger extent than in other populations. Our findings add evidence supporting the substantial contribution of *CYP3A5* genetic inter-individual variations to CYP3A-dependent drug clearance, unlike *CYP3A4*. Indeed, a study conducted among pregnant women from Tanzania reported no significant effects of *CYP3A4* genetic variability but found significant effects of the *CYP3A5* genotype on the plasma Lumefantrine concentration [15]. Several other studies have reported significant effects of the *CYP3A5* genotype on the plasma concentration of CYP3A substrates [30,31].

On the other hand, *CYP3A5* genetic variation did not significantly affect the 4β-OHC/Chol ratio among pregnant women, suggesting non-significant difference in CYP3A enzymes activity in pregnant women with zero or one functional genotype compared to those with two functional *CYP3A5* genotypes, as shown in Figure 3. Further analysis revealed a decrease in the differences of the geometric mean of the 4β-OHC/Chol ratio between pregnant women with two functional *CYP3A5* alleles and those with zero or one functional *CYP3A5* alleles from the second trimester (0.02) to the third trimester (0.01) (Figure 2). The lack of significant effects of *CYP3A5* genetic variation might be explained by the observed pregnancy induction effect on CYP3A enzymes [32].

As one of the limitations, this study enrolled pregnant women starting from the second trimester. Thus, the pregnancy induction effect on CYP3A enzymes activity was not assessed from the first trimester. However, the present study used a relatively large sample compared to previous studies in pregnant women.

## 4. Materials and Methods

### 4.1. Participant Recruitment, Sample Collection and Processing

A cohort of Tanzanian pregnant women (*n* = 110) in their second trimester was recruited during their first ANC visit. The gestational age at enrollment was determined by fundal height and last menstrual period, which is the standard of care in Tanzania. Pregnant women who visited their ANC for the first time were recruited for this study at the ANC clinic. Pregnant women in their third trimesters were excluded. Usually, women begin their first ANC late [33], thus only few were found in their first trimester and were also excluded. Non-pregnant women (*n* = 59) from the same population were recruited at the general outpatient clinic as a control group. Women who were on long-term treatment for chronic diseases (HIV, TB) and those taking medications known to affect drug-metabolizing enzymes were excluded. Participants who reported the use of grapefruit juice were also excluded.

At enrollment, 5 mL of venous blood was collected from all participants. Two mL of the collected blood was stored at −80 °C in EDTA tubes and was used to extract DNA for *CYP3A5* genotyping. The other 3 mL of whole blood was immediately centrifuged at 2000× *g* for 10 min to obtain plasma. Pregnant women were followed to their third trimesters when 3 ml venous blood sample was collected and centrifuged to obtain plasma. The obtained plasma was stored at −80 °C and was used for the determination of plasma 4β-OHC and Chol levels.

### 4.2. Determination of Plasma 4β-OHC Levels

Plasma 4β-OHC was extracted using a previously described method [25]. Briefly, 250 μL of plasma samples and 50 µL (100 ng) of [^2^H_7_]4β-OHC (internal standard) were added to 15 mL glass tubes and briefly vortexed. To the mixture, 1 mL of 0.7 M ethanolic potassium hydroxide, 20 µL (10 mg/mL) of EDTA and 10 µL (5 mg/mL) of butylated hydroxytoluene (BHT) were added. The mixture was then kept at room temperature for 30 min to allow the hydrolysis of 4β-OHC esters. After 30 min, 160 µL of dilute phosphoric acid was added to lower the pH to about 7. The mixture was then centrifuged at 1000× *g* for 10 min, and the supernatant was loaded onto a solid-phase extraction column (Strata X, 30 mg, Phenomenex) previously washed with methanol and water. The supernatant was passed through the column, and the column was then washed with 1 mL (10%) of methanol in water. Subsequently, 4β-OHC was eluted with 1 mL of (85%) acetonitrile in water. The solvent was evaporated under nitrogen gas, and 100 μL of tert-butyldimethylsilylimidazole diformamide was added to convert 4β-OHC into its tert-butyldimethylsilyl ether derivative. The mixture was left at room temperature for 24 h and then incubated for 2 h at 65 °C. The 4β-OHC derivative was then extracted twice with isooctane, and the solvent was evaporated. Finally, the residue was dissolved in 60 µL of *n*-hexane and analyzed by gas chromatography–mass spectrometry (GC-MS).

The sample (3 µL) was injected into a Hewlett Packard HP6890 gas chromatographer connected to a Hewlett Packard HP 5973 mass selective detector. The gas chromatographer was equipped with an HP5 MS column (5% Phenylmethyl Siloxane, 30 m × 0.25 mm, 0.25 µm film thickness). The temperature program started at 180 °C, increased at 35 °C/min until 270 °C, then the rate was changed to 20 °C/min until a final temperature of 310 °C was reached, which was maintained for 20 min. The injector temperature was 250 °C, and the detector temperature was 280 °C. The helium carrier gas flow rate was 1 mL/min. The ions *m*/*z* 573 and *m*/*z* 580 were used for the detection of 4β-OHC and [^2^H_7_]- 4β-OHC, respectively. The standard curve was linear from 3 ng/mL to 600 ng/mL. With the requirements S/N > 5, precision better than 20%, accuracy of 80–120%, it was found that the LLOQ for 4β-OHC was 3 ng/mL. The within-day variation was 4.5% (*n* = 16), and the between-day variation was 8.2% (*n* = 59) at 25 ng/mL.

### 4.3. Determination of Plasma Cholestrol Levels

Plasma cholesterol was determined by gas chromatography–mass spectrometry. In brief, 5 µL of plasma and 2 µg of internal standard ([^2^H_6_] cholesterol) were added to a 15 mL glass tube and briefly vortexed. Then, 1 mL of ethanolic sodium hydroxide was added to the mixture, which was incubated at 65 ^°^C in a shaking water bath for 2 h. To the mixture, 0.5 mL of water was added. Cholesterol was extracted twice with 3 mL of cyclohexan. After evaporation of the solvent, 200 µL of pyridine/hexamethyldisilazane/chlorotrimethylsilane (3/2/1, *v*/*v*/*v*) was added to derivatize cholesterol into its trimethylsilyl ether derivative. After vortexing, the mixture was incubated at 65 °C for 30 min. The solvent was evaporated under nitrogen gas, and the residue was dissolved in 250 µL of *n*-hexane and transferred to GC–MS vials for analysis.

The sample (2 µL) was injected into an Agilent 6890 N gas chromatographer connected to an Agilent 5973 mass selective detector. The gas chromatographer was equipped with an HP-Ultra1 MS column (Methyl Siloxane, 25m × 0.20 mm, 0.33 µm film thickness). The temperature program started at 180 °C, increased at a rate of 20 °C/min until 250 °C, then the rate was changed to 35 °C/min until a final temperature of 300 °C was reached, which was maintained for 7.5 min. The injector temperature was 250 °C, and the detector temperature was 280 °C. The helium carrier gas flow rate was 1 mL/min. The ions *m*/*z* 458 and *m*/*z* 464 were used for the detection of cholesterol and [^2^H_6_] cholesterol, respectively. The standard curve was linear between 0.13 and 6.2 mmol/L. The between-day variation was 6.7% (*n* = 80) at 5.34 mmol/L.

### 4.4. Genotyping

Genomic DNA was isolated from whole-blood samples using the QIAamp DNA Midi Kit (Qiagen GmbH, Hilden, Germany). Genotyping for CYP3A5*3, CYP3A5*6, CYP3A5*7 was conducted as previously described [15]. Briefly, genotyping was performed on the 7500 Real-Time PCR system (Applied Biosystems) using TaqMan drug metabolism genotyping assay reagents for allelic discrimination (Applied Biosystems Genotyping Assays) with the following ID numbers for each SNP: C_26201809_30 for CYP3A5*3 (c.6986A4G, rs776746), C_30203950_10 for CYP3A5*6 (g.14690G4A, rs10264272), and C_32287188_10 for CYP3A5*7. The final volume of each reaction was 10 μL, consisting of 9 μL of TaqMan fast advanced master mix (Applied Biosystems, Waltham, MA, USA), TaqMan 20X drug metabolism genotyping assays mix (Applied Biosystems) and 1 μL of genomic DNA. The PCR profile consisted of an initial step at 60 °C for 30 s, a hold stage at 95 °C for 10 min and a PCR stage for 40 cycles, step 1 at 95 °C for 15 s, and step 2 at 60 °C for 1 min, and after a read stage at 60 °C for 30 s.

### 4.5. Data and Statistical Analysis

Based on previous data [12], an increase of 30% in mean 4β-OHC/Chol ratio in pregnant women in the third trimester as compared to non-pregnant women was assumed. The sample size was calculated on 80% statistical power at the 2-sided 5% level of significance and 95% confidence interval (CI). Descriptive analysis for categorical variables is presented using frequency (*n*, %). The median (interquartile range) was used to describe plasma cholesterol, 4β-OHC concentrations, and the 4β-OHC/Chol ratio. The comparison of median plasma cholesterol concentrations, 4β-OHC concentrations and 4β-OHC/Chol ratios between the second and the third trimesters in pregnant women was performed using the Wilcoxon Signed-Rank Test, whereas the comparison between pregnant and non-pregnant women was perforemd the using the Mann–Whitney U test. Variables in pregnant women in the second and third trimesters were compared separately to the corresponding variables in non-pregnant women. The percent changes in the plasma 4β-OHC/Chol ratio from the second to the third trimester were calculated using the following formula:

$$\%\;\mathrm{Change}\;\mathrm{in}\;4\beta -\mathrm{OHC}/\mathrm{Chol}\;\mathrm{ratio}=\;[\frac{\frac{4\beta \mathrm{OHC}}{\mathrm{Chol}}\;\mathrm{at}\;\mathrm{the}\;\mathrm{third}\;\mathrm{trimester}\;-\frac{4\beta \mathrm{OHC}}{\mathrm{Chol}}\;\mathrm{at}\;\mathrm{the}\;\mathrm{second}\;\mathrm{trimester}}{\frac{4\beta \mathrm{OHC}}{\mathrm{Chol}}\;\mathrm{at}\;\mathrm{the}\;\mathrm{second}\;\mathrm{trimester}\;}]\;\times \;100$$

i.e., % Change in 4β-OHC/Chol ratio = [(4β-OHC/Chol at the third trimester-4β-OHC/Chol at the second trimester)/(4β-OHC/Chol at the second trimester )] × 100

The plasma 4β-OHC/Chol ratio was log10-transformed before the statistical analysis using a parametric test. Repeated-measure ANOVA was used to examine the change of the log10 4β-OHC/Chol ratio between the second and the third trimesters and the effect of the *CYP3A5* genotypes on the log10 4β-OHC/Chol ratio among pregnant women. ANOVA and linear regressions were used to explore the effect of the *CYP3A5* genotypes on the log10 4β-OHC/Chol ratio in non-pregnant women. Statistical analyses were performed using IBM SPSS Statistics for Windows, Version 27.0 (Armonk, NY: IBM Corp). Graph Pad Prism version 8.3 for Windows (Graph Pad, La Jolla, CA, USA) was used for the graphical presentations; *p* values < 0.05 were statistically significant.

## 5. Conclusions

Our study showed that pregnancy increases CYP3A enzymes activity from the second to the third trimester. Furthermore, the *CYP3A5* genotype is a potential determinant of the 4β-OHC/Chol ratio in non-pregnant women and should be considered in studies involving black African populations.

## Figures and Tables

**Table 1:** Baseline characteristics of the study participants.

Variables		Pregnant Women (*n* = 110)	Non-Pregnant Women (*n* = 59)
Median age (IQR) (years)		27 (18 to 33)	35 (24 to 41)
Median weight (IQR) (kg)		55 (50 to 62)	65 (54 to 75)
Median gestational age (IQR) (weeks)		22 (19 to 24)	-
Gravidity *n* (%)	Primigravida	29 (26.4)	-
	Secundigravida	27 (24.5)	-
	Multigravida	54 (49.1)	-

**Table 2:** Levels of 4β-OHC, Chol and 4β-OHC/Chol ratio during pregnancy.

Variables		N	Median	IQR	Change in Median Value (%)	*p*-Value
4β-OHC (ng/mL)	Second trimester	110	30.25	24.62–40.60	9.43	0.003
	Third trimester	110	34.80	29.30–46.50		
Cholesterol (mmol/L)	Second trimester	110	4.04	3.30–5.32	2.84	0.82
	Third trimester	110	4.25	3.39–5.10		
4β-OHC/Chol × 10^4^ molar ratios	Second trimester	110	0.20	0.14–0.25	7.31	0.02
	Third trimester	110	0.22	0.15–0.32		

**Table 3:** Comparison of median plasma 4β-OHC, Chol and 4β-OHC/Chol ratio between non-pregnant women and pregnant women in their second and third trimesters.

Variable	N	Pregnant Women in Second Trimester	N	Non-Pregnant Women	*p*-Value	Pregnant Women in Third Trimester	*p*-Value
Median 4β-OHC (IQR) (ng/mL)	110	30.25 (24.62 to 40.60)	59	23.19 (17.94 to 29.49)	<0.001	34.80 (29.30 to 46.50)	<0.001
Median Chol (IQR) (mmol/L)	110	4.04 (3.30 to 5.32)	59	4.24 (3.74 to 4.81)	0.54	4.25 (3.39 to 5.10)	0.96
Median 4β-OHC/Chol × 10^4^ molar ratios (IQR)	110	0.20 (0.14 to 0.25)	59	0.14 (0.11 to 0.18)	<0.001	0.22 (0.15 to 0.32)	<0.001
Geometric mean 4β-OHC/Chol × 10^4^ molar ratios	110	0.20	59	0.14	<0.001	0.22	<0.001

## Data Availability

All the data analyzed in this study are presented in this manuscript.
